# Metagenomic Association Analysis of Gut Symbiont *Limosilactobacillus reuteri* Without Host-Specific Genome Isolation

**DOI:** 10.3389/fmicb.2020.585622

**Published:** 2020-11-16

**Authors:** Sein Park, Martin Steinegger, Ho-Seong Cho, Jongsik Chun

**Affiliations:** ^1^Interdisciplinary Program in Bioinformatics, Seoul National University, Seoul, South Korea; ^2^Institute of Molecular Biology and Genetics, Seoul National University, Seoul, South Korea; ^3^School of Biological Sciences, Seoul National University, Seoul, South Korea; ^4^Laboratory of Swine Diseases, College of Veterinary Medicine and Bio-Safety Research Institute, Jeonbuk National University, Iksan, South Korea

**Keywords:** *Limosilactobacillus reuteri*, metagenome, pan-genome, host-specificity, host-symbiont interaction

## Abstract

*Limosilactobacillus reuteri* is a model symbiont that colonizes the guts of vertebrates in studies on host adaptation of the gut symbiont. Previous studies have investigated host-specific phylogenetic and functional properties by isolating the genomic sequence. This dependency on genome isolation is a significant bottleneck. Here, we propose a method to study the association between *L. reuteri* and its hosts directly from metagenomic reads without strain isolation using pan-genomes. We characterized the host-specificity of *L. reuteri* in metagenomic samples, not only in previously studied organisms (mice and pigs) but also in dogs. For each sample, two types of profiles were generated: (1) genome-based strain type abundance profiles and (2) gene composition profiles. Our profiles showed host-association of *L. reuteri* in both phylogenetic and functional aspects without depending on host-specific genome isolation. We observed not only the presence of host-specific lineages, but also the dominant lineages associated with the different hosts. Furthermore, we showed that metagenome-assembled genomes provide detailed insights into the host-specificity of *L. reuteri*. We inferred evolutionary trajectories of host-associative *L. reuteri* strains in the metagenomic samples by placing the metagenome-assembled genomes into a phylogenetic tree and identified novel host-specific genes that were unannotated in existing pan-genome databases. Our pan-genomic approach reduces the need for time-consuming and expensive host-specific genome isolation, while producing consistent results with previous host-association findings in mice and pigs. Additionally, we predicted associations that have not yet been studied in dogs.

## Introduction

*Limosilactobacillus reuteri* is a Gram-positive bacterial symbiont that has been recently reclassified from *Lactobacillus reuteri* (Zheng et al., 2020). This species colonizes the gut in a variety of vertebrate species and is used as a model organism to study the evolutionary process of vertebrate gut symbionts (Oh et al., 2010; Walter et al., 2011). The evolutionary trajectories of *L. reuteri* have previously been studied through amplified-fragment length polymorphism, multi-locus sequence analysis, and core-genome phylogeny (Oh et al., 2010; Wegmann et al., 2015; Yu et al., 2018). These studies identified genetically distinct subpopulations that highly correlate with their host, indicating a stable host-symbiont relationship. However, some outliers were also found wherein the strains from unrelated hosts were included in these host-specific clusters, which suggested occasional horizontal transfer between hosts (Oh et al., 2010; Walter et al., 2011). The transfer from one individual host to another could simultaneously occur in different host populations, resulting in distinct phylogenetic lineages, even in the same host species (Wegmann et al., 2015).

The adaptation of *L. reuteri* to the respective host resulted in host-specific functional features. For example, comparative genomics analysis of isolates identified host-specific genes with functions related to transposable elements and biofilm formation (Frese et al., 2011; Duar et al., 2017; Yu et al., 2018), which has been experimentally verified in mice (Frese et al., 2011, 2013; Duar et al., 2017).

However, analysis based on the isolated strains might not be representative of the complete repertoire of host-associated features of *L. reuteri* because isolating and sequencing a single bacterial strain under appropriate culture conditions remains challenging (Teeling and Glockner, 2012). Currently, there are 151 isolated genomes (April 2020) available in the EzBioCloud database (Yoon et al., 2017a), but the majority originate from well-studied model species, such as rodents, humans, pigs and poultry, limiting our ability to study a wider range of host adaptations.

**FIGURE 1:**
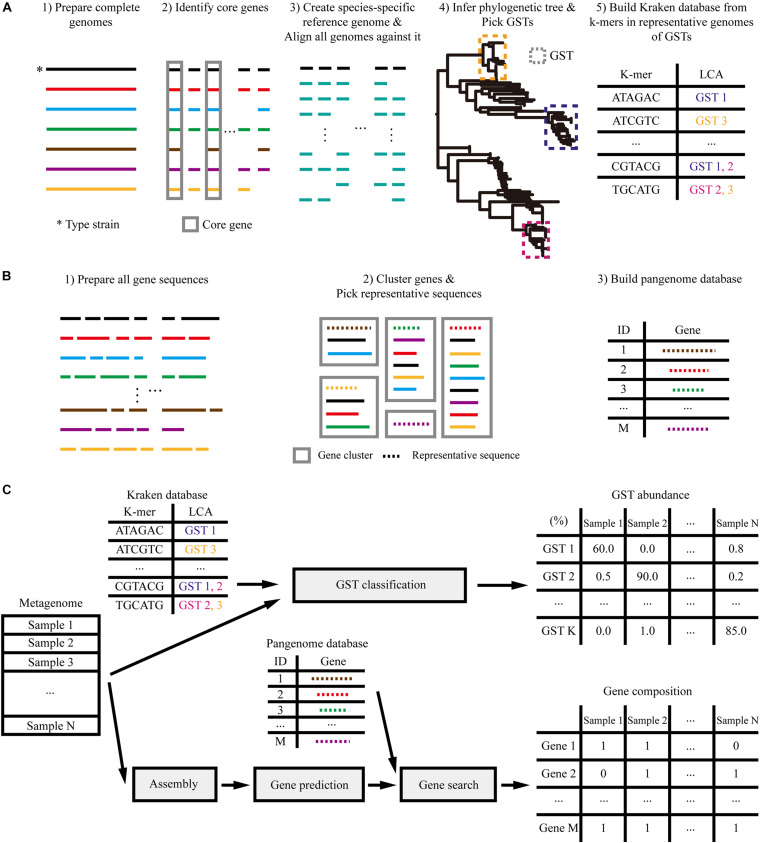
Overview of analysis. **(A)** Construction of the Kraken database of genome-based strain types (GSTs) based on the phylogenetic tree. **(B)** Construction of the pan-genome database. **(C)** Profiling the metagenome samples based on the reference databases built above.

In this study, we devised a method (Figure 1) to analyze the association of *L. reuteri* with hosts in metagenomes that overcome the need for host-specific genome isolation and successfully applied to gut microbiome samples of three mammals: pig, mouse, and dog.

## Results

### Construction of Genome-Based Strain Types (GSTs) and Gene Composition Profiles to Characterize Metagenomic Samples

To predict the phylogenetic features of *L. reuteri* in metagenome samples, we profiled the composition of GSTs. For this, a species-specific reference genome, phylogenetic tree and Kraken (Wood and Salzberg, 2014) database were built using complete genomes from the EzBioCloud database (Yoon et al., 2017a; see Supplementary Table 1), as described in Figure 1A. We created the *L. reuteri* reference genome by concatenating 1,158 core genes from eight complete genomes identified by Roary (Page et al., 2015), resulting in a 1,097,896 bp sequence. All 151 available *L. reuteri* (as of April 2020) strains isolated from humans, rodents, pigs, poultry, herbivores (goats, sheep, cows, and horses), and food sources (Supplementary Table 1) were aligned against the reference genome using MUMmer (Kurtz et al., 2004). The resulting multiple sequence alignments were used to infer a maximum likelihood phylogenetic tree using RAxML (Stamatakis, 2014). We then clustered the tree into 20 types by merging adjacent clades until a maximum all-against-all pairwise distance of 20,000 single nucleotide variations (SNVs) was reached. A reference Kraken database was built using core gene sequences from representative genomes of each type (Supplementary Figure 1).

The GSTs from our phylogenetic tree were in correspondence with previously reported host-associated lineages (Duar et al., 2017; Yu et al., 2018), and we could assign the GSTs to those lineages based on the tree in Supplementary Figure 2. GST 1, 12, 13, 15, and 16 were matched to “Human II/Herbivore,” “Porcine IV,” “Human VI/Poultry VI,” “Herbivore” and “Porcine V” lineages, respectively. GST 5 to 9 and GST 17 to 20 were assigned to “Rodent I” and “Rodent III” lineages, respectively, which were also found to be highly heterogeneous in the past work (Oh et al., 2010). The remaining GSTs were unassigned since they could not form a monophyletic group with others that corresponded with the host-associated lineages.

Moreover, we inferred functional features of *L. reuteri* based on gene composition profiles, which indicated the absence and presence of genes in each sample. As described in Figure 1B, a reference pan-genome database was constructed for *L. reuteri* using the coding sequences (CDSs) from 151 genomes. The database was comprised of a collection of 20,014 *L. reuteri*-specific gene clusters, which were obtained using 90% DNA similarity and 90% alignment coverage threshold. These clusters included 1,149 core ones found in over 95% of the genomes and 8,926 singletons.

These reference databases based on isolated genomes were used to characterize the metagenome samples by GST abundance and gene composition (Figure 1C). We profiled each sample by searching its reads against our GST database using Kraken (Wood and Salzberg, 2014) and estimated the abundance using Bracken (Lu et al., 2017). The gene composition was profiled through assembly using MEGAHIT (Li et al., 2015), genes were predicted using Prodigal (Hyatt et al., 2010) and annotated using MMseqs2 (Steinegger and Soding, 2017).

### Evaluation of Profile Estimation Using Synthetic Samples

We evaluated the accuracy of GST classification at the read-level and the composition level using synthetic samples. The synthetic samples were created using InSilicoSeq (Gourle et al., 2019) with three different complexity levels: four low complexity, four middle complexity and two high complexity, containing either five or 10 randomly selected GSTs or all 20 types, respectively. The precision at the read-level, defined as the proportion of correct assignments in GST and its ancestors to the total number of assignments, achieved an average precision of 95.68%, 95.16%, and 92.39% in the low, middle and high-complexity samples, respectively (Supplementary Table 2). We also measured the accuracy of the composition-level classification by computing Pearson’s correlation coefficient between the estimated and true abundance, obtaining 0.9937, 0.9879, and 0.9729 on average in the low, middle and high-complexity samples, respectively (Supplementary Figure 3).

Moreover, the accuracy of gene composition profiling was measured using the true positive rate (TPR) and F1 score. We simulated metagenomic samples from 12 reference genomes using InSilicoSeq (Gourle et al., 2019; see Supplementary Figure 4A legend) at four different coverage levels (1×, 5×, 10×, and 20×) and constructed gene profiles from these samples. We obtained TPRs ≥ 75% at 5× coverage and ≥90% at 10× and 20× coverage (Supplementary Figure 4A), and F1 scores of ≥80% at 5× coverage, and ≥90% at 10× and 20× coverage (Supplementary Figure 4B). In the case of the real metagenomic samples, coverage was 23.38× on average, with a standard deviation of 22.41 (Supplementary Figure 4C).

### Genome-Based Strain Typing to Characterize Host-Associative *L. reuteri* Populations in Real Metagenomic Gut Samples

**FIGURE 2:**
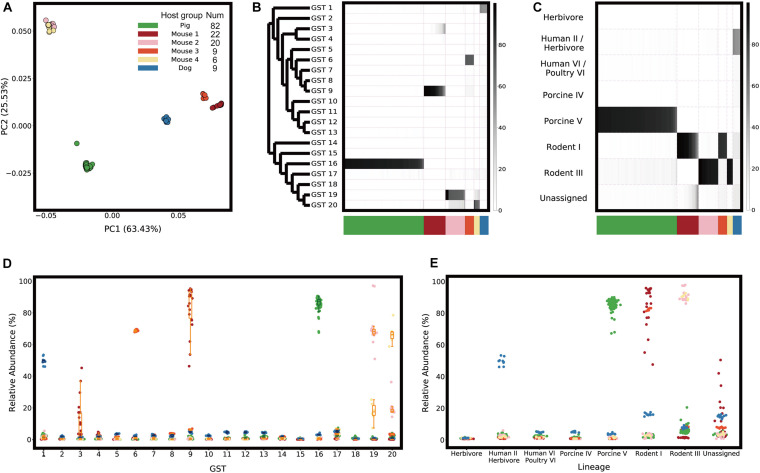
GST abundance profiles. **(A)** Population structures of *L. reuteri* in metagenome samples are visualized as Principal Coordinate Analysis (PCoA) plots with weighted UniFrac distance metric. **(B)** GST abundance profiles are represented as a heatmap, visualizing the relative abundance of the GSTs. Phylogenetic relationships between the GSTs are illustrated as a tree on the left (branch lengths are ignored). **(C)** A heatmap visualizing the relative abundance of host-specific lineages assigned from the GSTs. The host groups are shown in different colors on the bottom. Scatter and box plots representing the relative abundance of **(D)** GSTs and **(E)** host-specific lineages of each host group and sample.

Using the GST abundance profiles created from the real metagenomic gut samples, the samples were found not to contain a mixture of similarly distributed GSTs but a few abundant types, which showed an association with the host origin (Figure 2).

Since distinct host-specific lineages could be found from the same host origin, the host species were divided into “host groups” based on the most abundant type (“dominant GST”) in the metagenomic samples. We identified six host groups: one “Pig” group, four “Mouse” groups, and one “Dog” group (Figure 2A). The dominant GSTs of each host group were GST 16 in “Pig” samples (*n* = 82), GST 9 in “Mouse 1” samples (*n* = 22), GST 19 in “Mouse 2” samples (*n* = 20), GST 6 in “Mouse 3” samples (*n* = 9), GST 20 in “Mouse 4” samples (*n* = 6), and GST 1 in “Dog” samples (*n* = 9) (Figures 2B,D). Except for the dog samples, for which isolated genome sequences were unavailable in the reference database, the host group of the metagenome samples was associated with the isolation sources of the dominant GSTs of the samples (Supplementary Figure 1).

**FIGURE 3:**
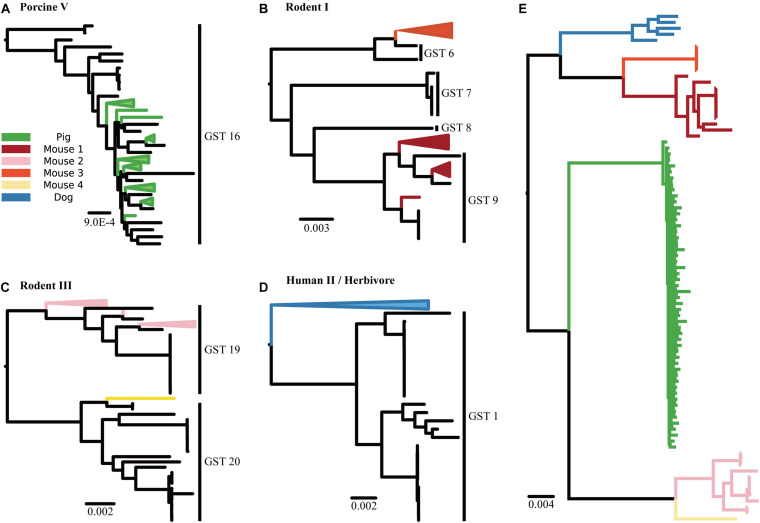
Phylogenetic trees of MAGs. Phylogenetic trees display the placement of *L. reuteri* MAGs assembled from the reads of the metagenome samples in the **(A)** “Pig” group, **(B)** “Mouse 1” and “Mouse 3” groups, **(C)** “Mouse 2” and “Mouse 4” groups and **(D)** “Dog” group, respectively. **(E)** A phylogenetic tree inferred from the MAGs without reference genomes.

These dominant GSTs were consistently found in the placement of 85 medium-to-high quality metagenome-assembled genomes (MAGs) in the phylogenetic tree (Figures 3A–D). Unlike the GST profiles, the MAG placements represented the phylogenetic relationship between *L. reuteri* in the samples and reference strains. For example, the MAGs from the pig samples were placed into the GST 16 clade (Figure 3A), whereas those from the dog samples formed their own clade outside the reference GST 1 clade (Figure 3D). These MAGs also showed a clear separation by the host groups in the phylogenetic tree, even without the reference tree of isolated genomes (Figure 3E).

The relative abundance of the dominant GSTs was different in each host group: the median abundance in “Pig” samples, “Mouse 1” to “Mouse 4” samples, and “Dog” samples was 87%, 90%, 68%, 68%, 66%, and 50%, respectively (Figure 2D). This abundance indicated that a single dominant GST occupied more than half of the *L. reuteri* population, despite the variation in abundance. However, we also found non-dominant GSTs with relative abundance above 10%. “Mouse 2” and “Mouse 4” samples contained 18% of GST 20 and 17% of GST 19, respectively. Not only the isolation sources of the dominant GSTs, but the non-dominant GSTs coincided with the host groups of the samples (Supplementary Figure 1).

Furthermore, we assessed how distinct each *L. reuteri* population between host groups was by performing a permutational multivariate analysis of variance (PERMANOVA) (Anderson, 2001) test. This revealed that the GST abundance of the samples was significantly different from those of other samples included in the different host groups (Supplementary Figure 5).

### Functional Features of *L. reuteri* Associated With Host Origin

Phylogenetically, we measured the host association of *L. reuteri* using the GST profiles. Furthermore, we aimed to determine whether host specificity was reflected in the functional profiles. To investigate this, we selected host-specific genes from the gene composition profiles, which were created by searching metagenomic sequences against the pan-genome of the isolated strains.

**FIGURE 4:**
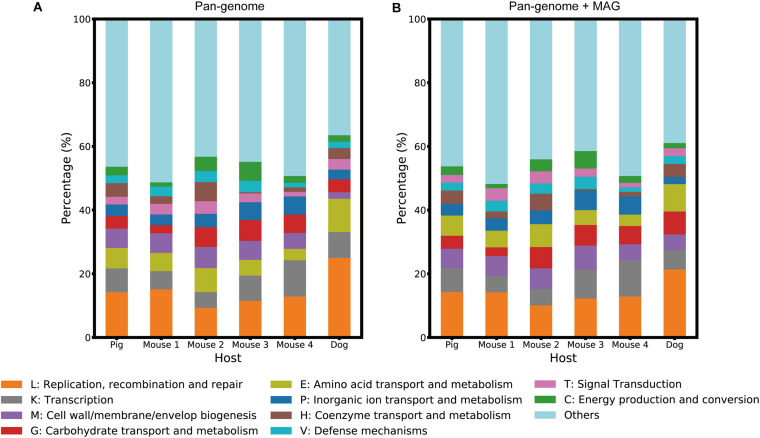
Host-specific functional structures of each host group. The stacked bar plots visualize the proportion of the functional categories of host-specific genes. **(A)** represents the host-specific genes identified from the pan-genome database, while **(B)** represents those identified from the pan-genome database and the MAGs.

We detected significant differences in *L. reuteri* gene composition between the host groups using the PERMANOVA test (Supplementary Figure 6). A set of 4,128 host-specific genes, including 2,172 “Pig,” 540 “Mouse 1,” 644 “Mouse 2,” 354 “Mouse 3,” 207 “Mouse 4” and 211 “Dog”-specific genes, were identified using Fisher’s exact test, and assigned to clusters of orthologous group (COG) (Tatusov et al., 2000) annotation (Figure 4A and Supplementary Table 3). These genes mainly belonged to four functional categories: (1) replication, recombination and repair, (2) transcription, (3) transport and metabolism of various macromolecules and ions, and (4) cell wall/membrane/envelope biogenesis.

Host-specific genes were obtained not only from the pan-genome database but also from MAGs. We predicted 132,255 CDSs from 85 MAGs and identified a set of 1,913 host-specific genes from them by performing Fisher’s exact test. Compared to the host-specific gene set identified from the pan-genome database, this MAG-based gene set contained 418 of newly found host-specific genes. These novel genes were annotated based on the eggNOG (Huerta-Cepas et al., 2019) database (Supplementary Table 4), and the proportion of COG functional categories of all host-specific genes was computed, as shown in Figure 4B. We compared the functional structures of these genes based on the pan-genome to those based on both pan-genomes and MAGs and found that the 10 most abundant categories were conserved despite some differences in ratios (Figure 4). However, if the host-specific reference genome was absent, a relatively high percentage of the host-specific genes were newly identified from the MAGs. Approximately 37% of dog-specific genes were exclusively found in the MAGs, while 2% of pig and 20% of mouse-specific genes were found only in the MAGs (Supplementary Table 5).

From the detailed functional description in Supplementary Tables 3, 4, transposases, integrases and ATP-binding cassette transporters were found to be host-specific in all host groups. However, some gene functions were not identified in all host groups; for example, host-specific urea amidohydrolases were observed only in the “Mouse 1” and “Mouse 2” groups. These host-specific functions, especially those related to mobile elements and biofilm formation, reflect differences in the host gut environment and the adaptation mechanism of *L. reuteri* to their host, which consistently supports the observations of previous studies with isolated strains (Frese et al., 2011, 2013).

## Discussion

We confirmed that our computational findings are consistent with previously published results based on genome isolation by comparing it in two ways: phylogenetic trees and host-specific genes. (1) As shown in Supplementary Figure 2, our phylogenetic tree is similar to a previously published tree (Duar et al., 2017; Yu et al., 2018), which makes it possible to assign our GSTs to host-specific lineages reported before and to profile metagenome samples with those lineages (Figures 2C,E). The GSTs could explain the phylogeny of *L. reuteri* population in the samples further by clustering highly heterogeneous lineages into several types based on fixed amounts of variation. For example, “Mouse 1” and “Mouse 3” samples mainly contained *L. reuteri* strains in “Rodent I” lineages but different GSTs, GST 9 and 6, respectively. (2) Host-specific genes identified from the gene profiles using Fisher’s exact test could be assigned to functions such as biofilm formation and mobile element, which were previously highlighted as host-specific functions of various hosts, such as mice (Frese et al., 2011, 2013), herbivores (Yu et al., 2018), and chickens (Duar et al., 2017).

Moreover, our method can detect novel host-specific patterns of *L. reuteri* even without the respective isolates in the reference database. We showed this by assigning dog samples to GSTs, the majority of *L. reuteri* strains within them were classified as GST 1 and their populations were significantly different from those in other host groups as determined using PERMANOVA test. GST 1 is distinctive to others since it consists of not just one dominant isolation source but two: human and herbivore. Placing MAGs from the dog sample in our phylogenetic tree demonstrated the presence of an independent clade of dog strains, which suggested that the divergence between human- and herbivore-specific lineages in reference GST 1 strains was preceded by the divergence of a dog-specific lineage. Mechanisms of these host transfer and divergence would possibly be caused by similar gastrointestinal conditions between the hosts, horizontal acquisition of new traits or dietary glycans, helping *L. reuteri* adapt in other hosts (Duar et al., 2017).

Unlike the MAG placement, the GST profiles based on read-mapping did not explain evolutionary trajectories; they only represented the relative abundance of GSTs in the samples. Nonetheless, analysis based on read-mapping has advantages over MAG placement-based analysis. First, obtaining high-quality MAGs requires a large number of samples (Alneberg et al., 2018). In this study, we obtained 85 medium-to-high quality MAGs from 148 metagenomic samples. Since the quality of MAGs depends on the quality of assembly (Sangwan et al., 2016; Alneberg et al., 2018), the phylogenetic placement would be inaccurate if the assembled contigs contained too much noise or had low coverage. Additionally, the MAG often represents multiple strains rather than an individual, which can cause a bias in the strain-level analysis (Sangwan et al., 2016; Alneberg et al., 2018).

Metagenome-assembled genomes also provided many novel functional features. Approximately 10% of the host-specific genes were novel in the MAGs, in that 4,128 and 418 of these genes were identified using Fisher’s exact test from the isolates and MAGs, respectively. In particular, if the host-specific reference genome is unavailable, such as for dogs in this study, a relatively high proportion of novel host-specific genes is found only based on MAGs. However, some limitations of using MAGs mentioned above can lead to incorrect genes and functional features. Therefore, additional genome isolation of the strains should be done continuously, and decontamination techniques for the MAGs need further improvement. Furthermore, statistical tests to select genes that are significantly specific to the host and to filter those that are exceptionally found in few samples are necessary for the functional analysis.

In summary, our approach utilized metagenomic reads instead of isolated bacterial genomes to analyze the host-symbiont association, allowing the application of this approach to the gut metagenome of any host, such as dogs in this study. We investigated the host-specific population structures and functional features of *L. reuteri* in metagenomic samples through the reference pan-genome and MAGs. In addition to demonstrating a consistent association with previously studied hosts, this approach could also be applied to a host with no existing isolated strains.

## Materials and Methods

### Prioritization of Reference Genomes

In total, 151 high-quality reference *L. reuteri* genomes from the EzBioCloud database (Yoon et al., 2017a) were prepared and sorted based on the level of genome assembly completeness, with complete genomes being prioritized followed by chromosome-level assembled genomes, then others. Those in the same assembly completeness level were re-sorted by their N50 values.

### Phylogenetic Analysis and Strain Typing of *L. reuteri*

The ML phylogenetic tree of *L. reuteri* was created through the workflow described by Ha et al. (2019). First, 14 complete reference genomes were selected, and UBCG core genes (Na et al., 2018) were extracted from each genome. The UBCGs for each complete genome pair were aligned to compute the similarity. The genomes with a median similarity of 100% in all pairs were grouped, and those with the highest priority in each group were selected as representative genomes. These eight representatives were used for *L. reuteri* core-genome identification. A set of core genes was generated by adding only those shared among the complete genomes of *L. reuteri* using the Roary v3.12.0 pipeline (Page et al., 2015). A species-specific reference genome for *L. reuteri* was then created by concatenating the core gene set of the type strain. Multiple sequence alignment was created from the SNVs of all 151 genomes aligned against the reference genome in this study using MUMmer v.3.23 (Kurtz et al., 2004). An ML tree was inferred by RAxML v.8.2.8 using the GTRCAT nucleotide model (Stamatakis, 2014).

The GSTs were defined as the largest clades, where the number of SNVs was less than 20,000 in all pairs of the clade members, clustered from *L. reuteri* genomes. Starting from the type strain genome, the sister clades were merged on the ML tree into the GST as long as the maximum number of SNVs between the clade members was below 20,000. The merging step was repeated until everything was clustered, starting from the genome with the highest priority. The genome with the highest priority of each type was selected as the representative.

Using the Kraken-build script in the Kraken software package (Wood and Salzberg, 2014), a database with a k-mer length of 31 was constructed from the core genes of the representative genomes. Since Kraken by default uses the NCBI taxonomy to assign k-mers to a taxonomic level, custom taxonomy was provided following the topology of the ML tree to map k-mers to the lowest common ancestor.

### Construction of the *L. reuteri* Reference Pan-Genome

To produce an *L. reuteri*-specific gene database, all CDSs from *Lactobacillus* were collected from the EzBioCloud database and clustered into orthologous groups using Linclust (Steinegger and Soding, 2018) at 90% sequence identity and 90% bi-directional coverage thresholds. Only representative sequences of the clusters that contained *L. reuteri* were added to the pan-genome database and assigned a COG based on the annotation in the EzBioCloud database (Yoon et al., 2017a).

### Metagenomic Sample Collection and Sequencing

Metagenomic sequence data were collected by not only downloading previously deposited data of pig, mouse, and dog gut microbiomes from the NCBI SRA database (Xiao et al., 2015; Rosshart et al., 2017; Coelho et al., 2018; Munk et al., 2018), but also by directly sequencing 20 pig samples (Supplementary Table 6).

All rectal grab fecal samples were collected aseptically from individual pigs on the same day at 75 days (30 kg) and 150 days (90 kg) of age. Total DNA from fecal samples was isolated using the FastDNA Spin Kit for Soil (MP Biomedicals), and its quality was checked using the Qubit dsDNA HS Assay Kit (Thermo Fisher Scientific). DNA libraries for whole-genome shotgun sequencing were prepared according to the Illumina TruSeq Nano protocol, and 2 × 100 paired-end sequencing was performed using an Illumina NovaSeq 6000 system.

### Metagenomic Sequence Data Processing

Species-level taxonomy profiles were created using a curated core gene-based bacterial database (Chalita et al., 2020). All samples not containing *L. reuteri* in their profiles were discarded.

Quality filtering and trimming were performed using BBDuk from the BBTools suite^1^. Adapter sequences and low-quality bases from termini at a quality threshold of Q12 were trimmed from the reads, followed by the removal of the reads with average quality below 10. All reads that passed quality control filtering were assembled into contigs using MEGAHIT v.1.1.3 (Li et al., 2015) with default k-mer sizes and a minimum contig length of 500 bp. Prodigal v.2.6.2 (Hyatt et al., 2010) was used in metagenomic mode to extract all genes from the assembly that were longer than 100 bp.

### Profiling GST Abundance and Gene Composition in the Metagenomic Samples

The reads in each metagenomic sample were mapped to the reference Kraken database, and the results were summarized using kraken-report. We used Bracken (Lu et al., 2017) to estimate GST abundance from the reports. The gene composition was profiled by searching the predicted genes from the assembled reads against the *L. reuteri* pan-genome using MMseqs2 (Steinegger and Soding, 2017) with 90% minimum sequence identity and bi-directional alignment coverage thresholds.

For the real metagenomic samples, those containing less than 20,000 reads classified as *L. reuteri* or with less than 500 predicted gene hits were removed. The remaining samples were clustered by their host group, which included the same dominant GSTs. The host group and its samples were filtered out if they did not include more than five samples.

### Metagenome Binning and Picking *L. reuteri* MAGs

MetaBAT2 v.2.14 (Kang et al., 2019) with the option “–minContig 1500” was used for contig binning. The coverage information was provided through Bowtie2 (Langmead and Salzberg, 2012), which mapped the reads into contigs and SAMtools (Li et al., 2009), which converted the mapping results into the BAM format. CheckM v.1.1.1 (Parks et al., 2015) was used to assess the completeness and contamination of each genome bin with UBCGs (Na et al., 2018) as the marker gene set, selecting medium-quality genomes as having completeness of ≥50% and contamination of <10%, and high-quality genomes with completeness of >90% and contamination of <5% (Bowers et al., 2017). The average nucleotide identity (ANI) values between the type strain genome of *L. reuteri* and MAGs were calculated using the OrthoANI tool (Yoon et al., 2017b), assigning the MAGs with an ANI value >95% to *L. reuteri* (Goris et al., 2007).

### Validation With Simulated Metagenome Data

Synthetic datasets were generated for three complexity levels from the *L. reuteri* reference genome sequences using InSilicoSeq v.1.3.5 (Gourle et al., 2019): (1) four low-complexity samples, (2) four middle-complexity samples, and (3) two high-complexity samples. The low, middle and high-complexity samples contained 10 million reads with five randomly selected GSTs, 50 million reads with 10 randomly selected GSTs, and 50 million reads with all 20 GSTs, respectively. The genome abundance was log-normally distributed in half of the samples and exponentially distributed in the remaining samples.

The read-level accuracy to identify *L. reuteri* GSTs in the metagenomic samples was assessed using a precision metric. The precision was computed by dividing the number of assignments to the correct type and its ancestors by the total number of assignments. The composition-level accuracy of the GST profiles was evaluated based on Pearson’s correlation coefficient. The coefficient between true and estimated GST abundance was calculated.

Furthermore, the TPRs and F1 scores were used to evaluate the accuracy of profiling *L. reuteri* gene composition. Overall, 12 complete isolated genomes were simulated into synthetic metagenome reads at four coverage levels: 1×, 5×, 10×, and 20×. These synthetic reads were assembled using MEGAHIT (Li et al., 2015), and CDSs were predicted from contigs ≥500 bp using Prodigal (Hyatt et al., 2010), as described above. Gene composition profiles for each simulated sample were constructed by searching CDSs ≥ 100 bp against the pan-genome database using MMseqs2 (Steinegger and Soding, 2017) with 90% minimum sequence identity and bi-directional alignment coverage thresholds. To compare the gene profiles to the true gene composition of isolated genomes, TPRs and F1 scores were defined as follows:

$$\mathrm{TPR}=\frac{\mathrm{true}\,\mathrm{positive}}{\mathrm{true}\,\mathrm{positive}+\mathrm{false}\,\mathrm{negative}}\,F_{1}=2\times \frac{\mathrm{precision}\times \mathrm{recall}}{\mathrm{precision}+\mathrm{recall}}$$

### Phylogenetic Placement and Novel Gene Identification of MAGs

Single nucleotide variations were detected from the medium and high quality *L. reuteri* MAGs using MUMmer (Kurtz et al., 2004), merged with the SNVs from 151 *L. reuteri* genomes into multiple sequence alignment. The MAGs were inserted into the phylogenetic tree of *L. reuteri* using RAxML with the “-f v” option (Stamatakis, 2014). Genes of the MAGs, predicted by Prodigal (Hyatt et al., 2010), were searched against the *L. reuteri* pan-genome database using MMseqs2 (Steinegger and Soding, 2017) and compared to the gene composition profiles of the corresponding metagenome samples to identify novel genes of MAGs. The unmatched genes were clustered using Linclust (Steinegger and Soding, 2018) with 90% of minimum sequence identity and bidirectional alignment coverage thresholds and annotated with eggNOG (Huerta-Cepas et al., 2019) assignments using eggNOG-mapper v.2.0.1 (Huerta-Cepas et al., 2017).

### Statistical Analysis

PERMANOVA (Anderson, 2001), with 9,999 permutations, was used to determine whether the GST abundance and gene composition of the samples from one host group were significantly different to those of another. Weighted UniFrac distance (Lozupone et al., 2011) for GST profiles and Jaccard distance for gene composition profiles were used as distance metrics.

For each host and gene, a contingency table representing the difference between the presence and absence of the genes in the gene composition profiles of the samples from the host was created and used for Fisher’s exact test to identify host-specific genes. The gene was found to be host-specific if the *p*-value was <0.01.

## Data Availability Statement

Raw metagenomic data are available from the SRA database with accession numbers PRJEB22062, PRJNA630862 for pig samples, PRJNA390686, PRJEB7759 for mouse samples and PRJEB20308 for dog samples. Codes for picking GSTs, building Kraken and pan-genome databases and profiling metagenomic data are available in https://github.com/psi103706/Lreuteri_strain_analysis.

## Author Contributions

SP implemented the software and performed the research. SP, MS, and JC designed the research and drafted the manuscript. H-SC generated the pig metagenomic data. All authors have read and approved the final manuscript.

## Conflict of Interest

The authors declare that the research was conducted in the absence of any commercial or financial relationships that could be construed as a potential conflict of interest.

## Footnotes

1 BBMap – Bushnell B. – sourceforge.net/projects/bbmap/
