# Supplementary material for: Metagenomic Association Analysis of Gut Symbiont Limosilactobacillus reuteri Without Host-Specific Genome Isolation
Source: Front Microbiol. 2020 Nov 16;11:585622. doi: 10.3389/fmicb.2020.585622 (PMC7717999; doi:10.3389/fmicb.2020.585622)
Supplement: Supplementary file 1 [file Data_Sheet_1.pdf]

Supplementary Material

1 Supplementary Figures

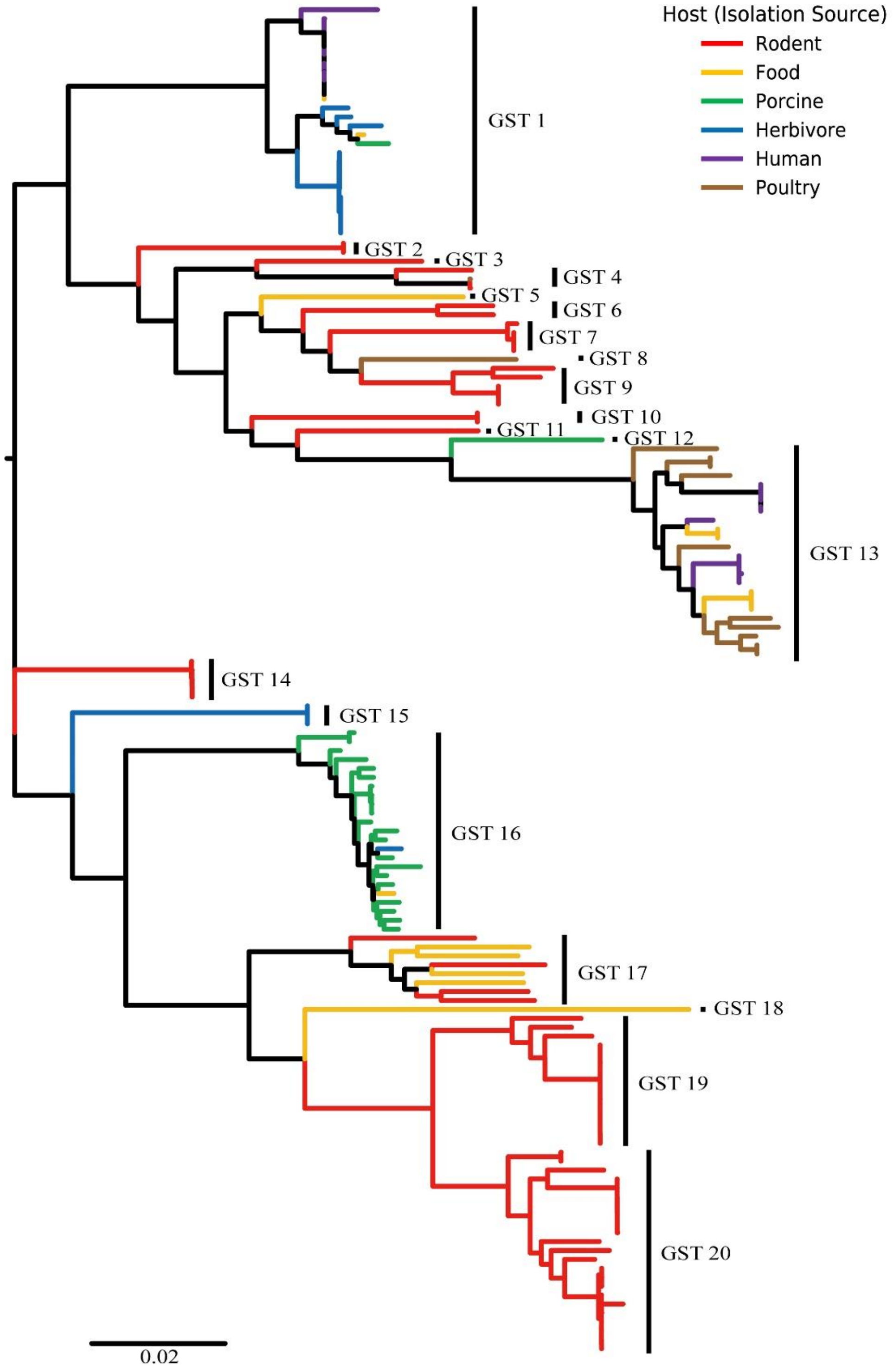

Supplementary Figure 1. Maximum likelihood phylogenetic tree of *L. reuteri* strains with GSTs and isolation sources.

The outgroups, *Limosilactobacillus vaginalis* ATCC 49540, *Limosilactobacillus panis* DSM 6035 and *Limosilactobacillus frumenti* DSM 13145, are not shown in the figure. The branches are colored by the isolation source of the strains.

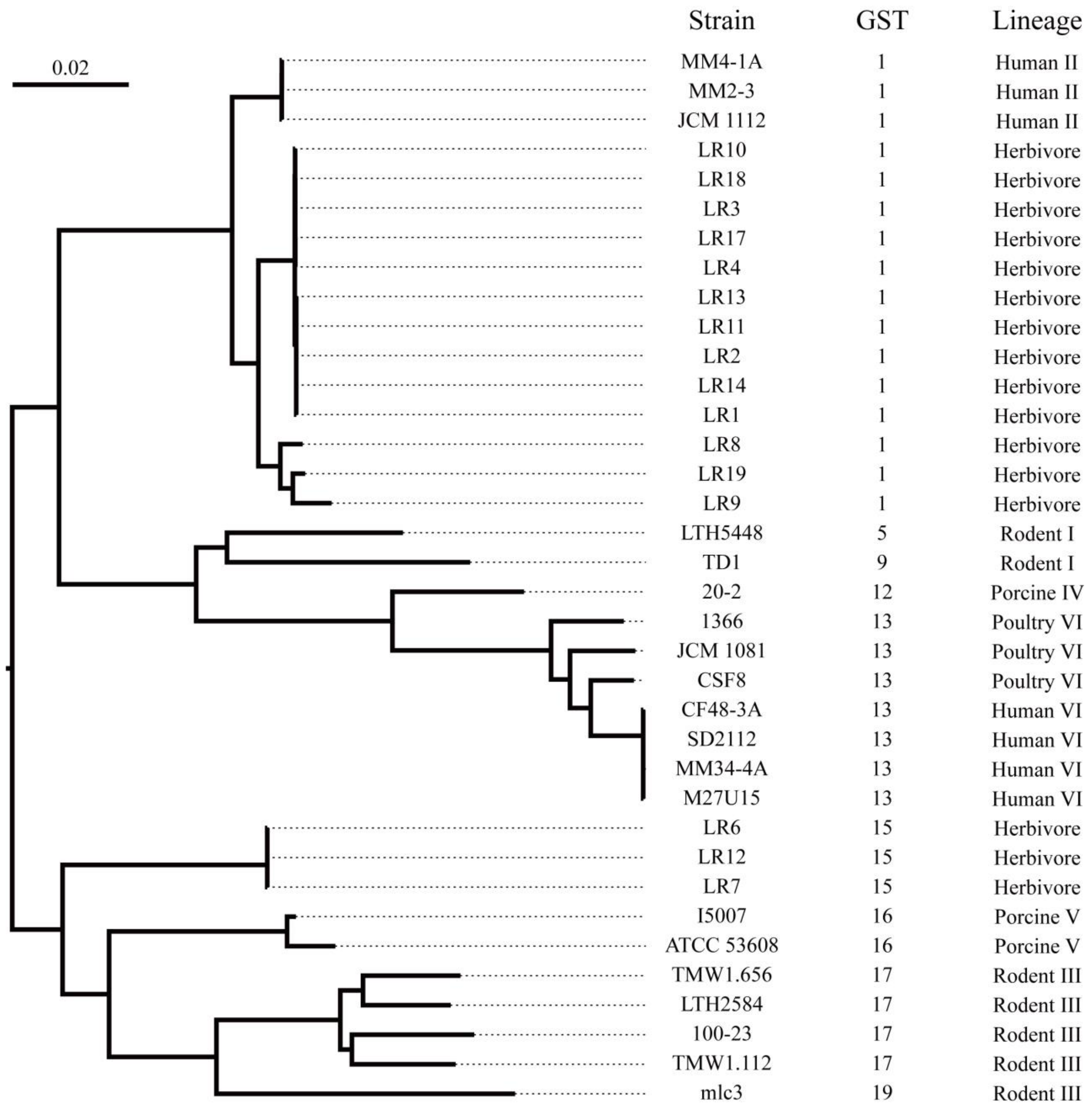

Supplementary Figure 2. Comparison of genome-based strain types (GSTs) and previously reported lineages (Duar et al., 2017; Yu et al., 2018).

Only the strains which existed in both studies were selected and represented on the phylogenetic tree.

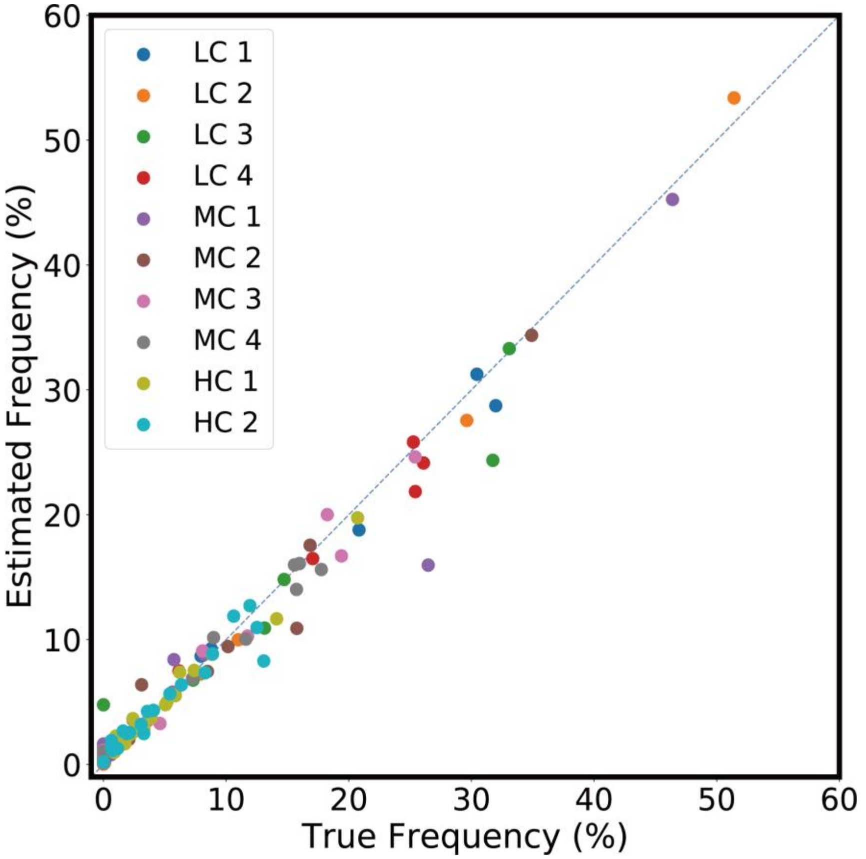

Supplementary Figure 3. Correlation between the estimated and true GST abundance of the synthetic samples. (LC: low complexity, MC: middle complexity, HC: high-complexity)

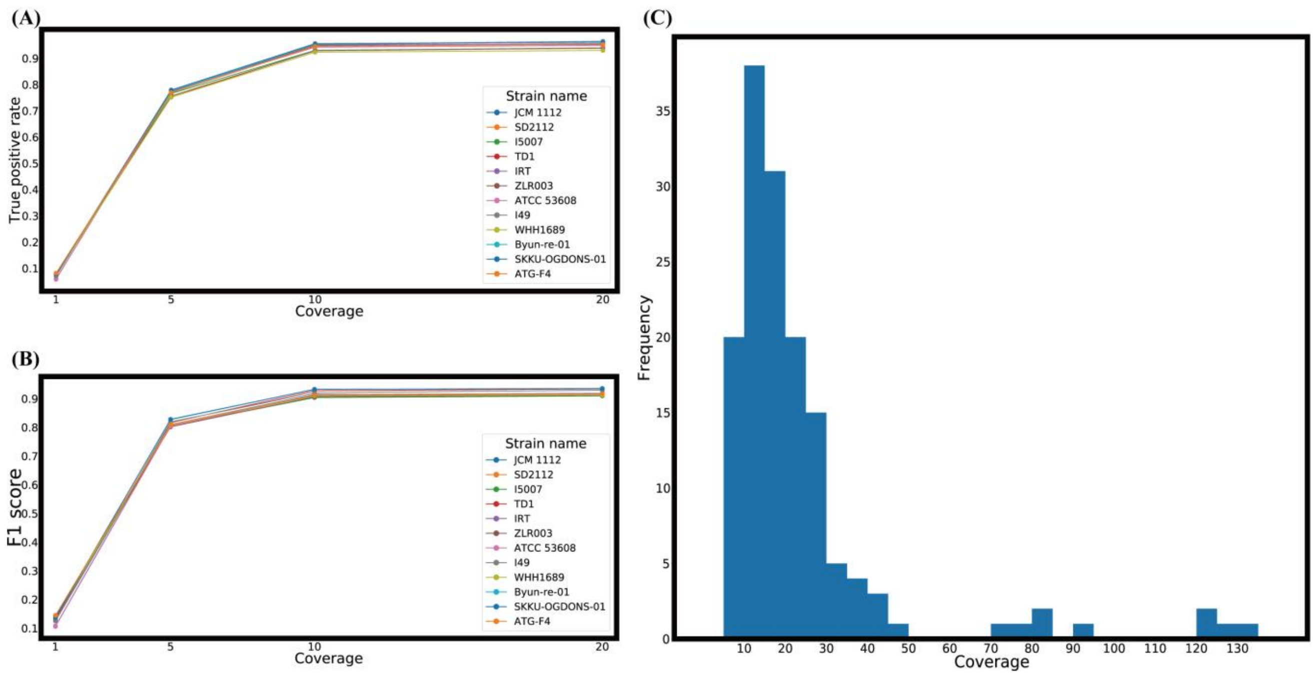

Supplementary Figure 4. The accuracy of gene search with respect to the coverage depth.

The plots report (A) true positive rate and (B) F1 score at the coverage of 1×, 5×, 10× and 20×, respectively. Synthetic data simulated from isolate genomes of twelve *L. reuteri* strains were used for the evaluation. (C) represents the distribution of the coverage level of the real metagenomic samples.

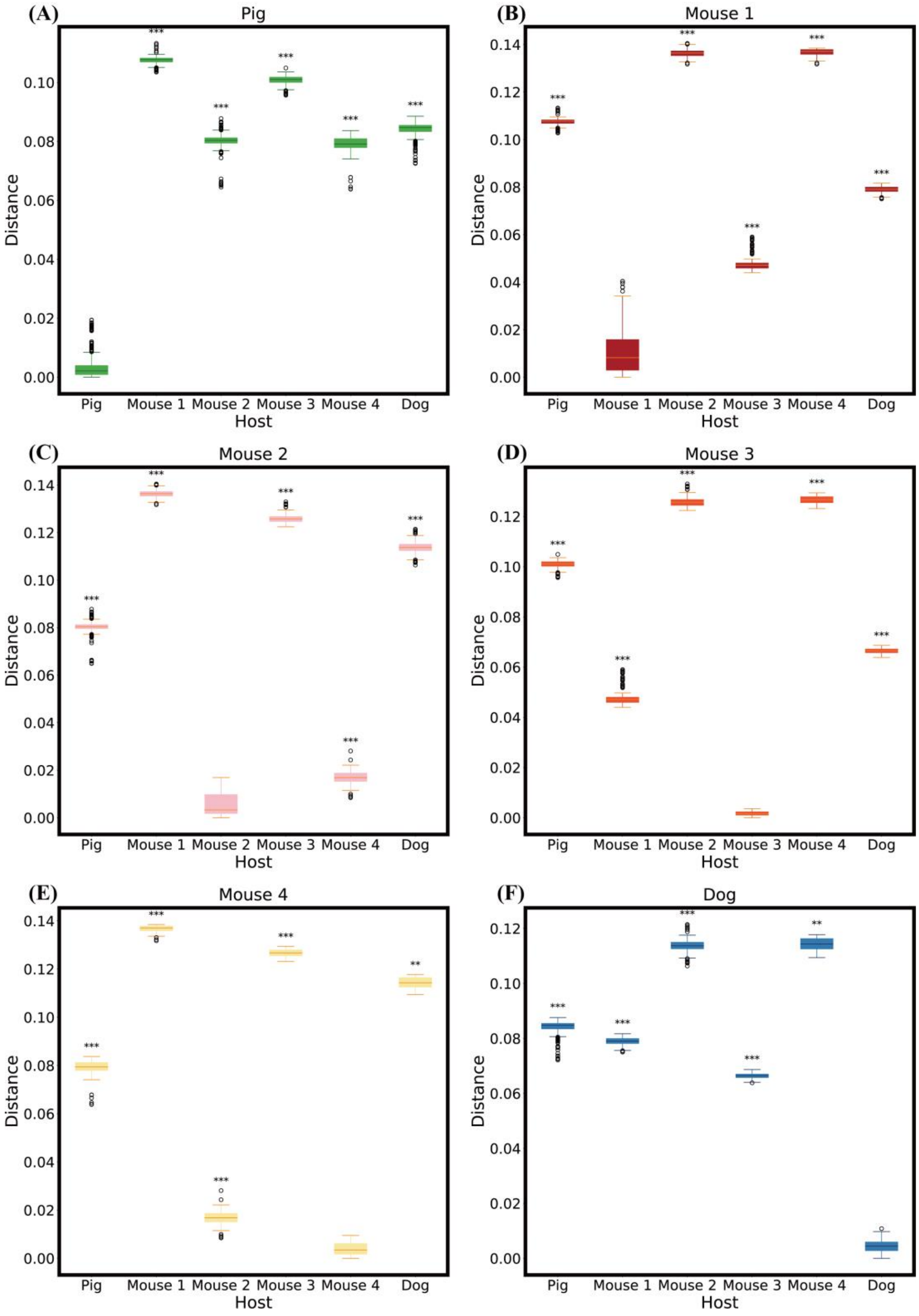

Supplementary Figure 5. Comparison of GST abundance using weighted UniFrac distance.

The plots represent the distance from (A) ”Pig” group, (B) “Mouse 1” group, (C) “Mouse 2” group, (D) “Mouse 3“group, (E) “Mouse 4“group and (F) “Dog” group, respectively. P-values of the PERMANOVA test were shown above each box in the plots (* : < 0.01, ** : < 0.001, ***: < 0.0001)

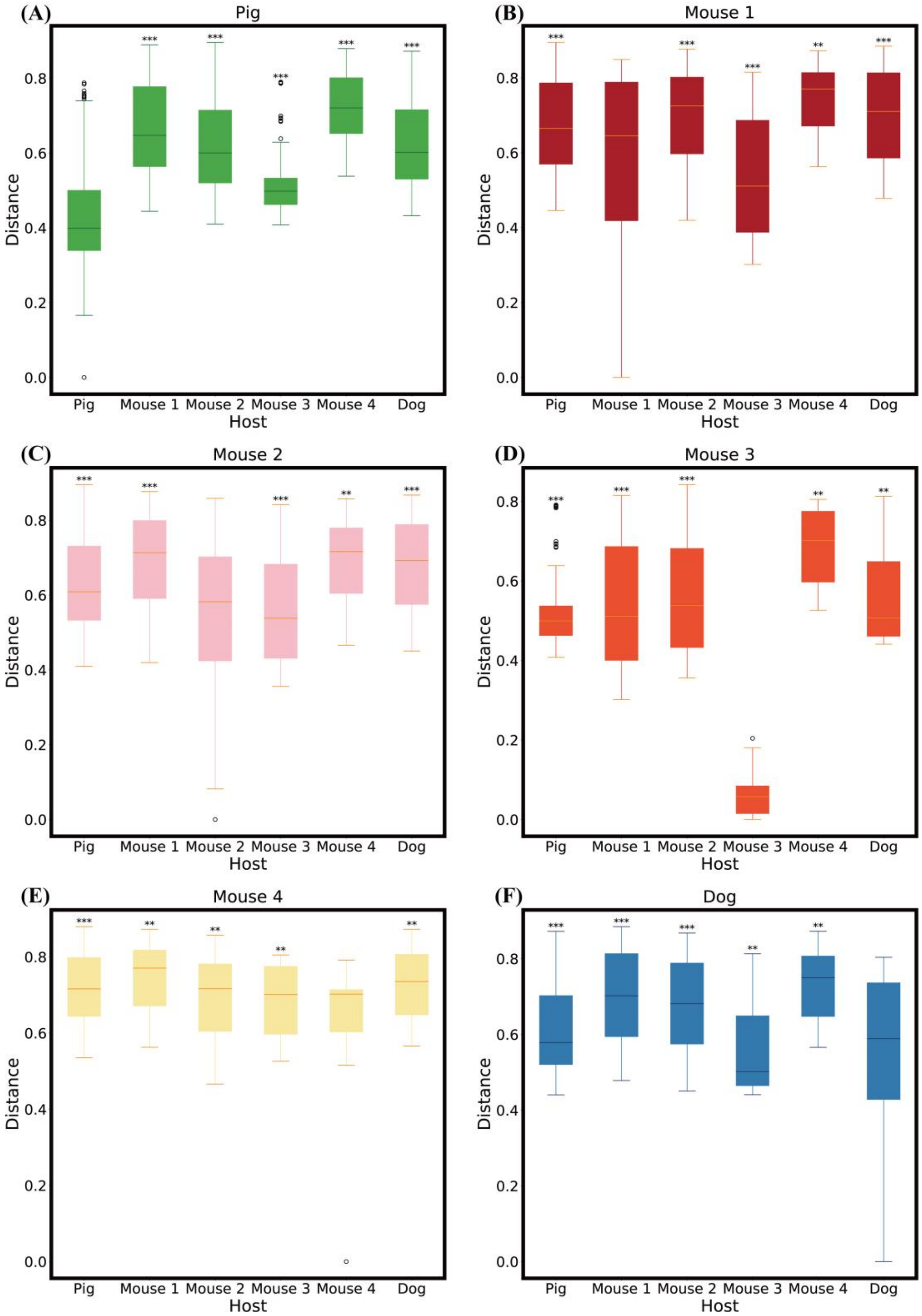

Supplementary Figure 6. Comparison of gene composition using Jaccard distance.

The plots represent the distance from (A) ”Pig” group, (B) “Mouse 1” group, (C) “Mouse 2” group, (D) “Mouse 3“group, (E) “Mouse 4“group and (F) “Dog” group, respectively. P-values of the PERMANOVA test were shown above each box in the plots (* : < 0.01, ** : < 0.001, ***: < 0.0001)
