# Supplementary material for: Metagenomic Association Analysis of Gut Symbiont Limosilactobacillus reuteri Without Host-Specific Genome Isolation
Source: Front Microbiol. 2020 Nov 16;11:585622. doi: 10.3389/fmicb.2020.585622 (PMC7717999; doi:10.3389/fmicb.2020.585622)
Supplement: Supplementary file 3 [file Data_Sheet_3.docx]

Supplementary Material

# Supplementary Tables

**Supplementary Table 1.** A list of isolated strains of *L. reuteri* used for the phylogenetic analysis.

**Supplementary Table 2.** The precision of read-level GST classification of the synthetic samples.

**Supplementary Table 3.** COG annotations and functional categories of host-specific genes identified by gene searching against the pan-genome database.

**Supplementary Table 4.** COG annotations and functional categories of host-specific genes identified only from the MAGs.

**Supplementary Table 5.** The number of host-specific genes found in the pan-genome database and MAGs from each host group.
